# The inactivation of the Niemann Pick C1 cholesterol transporter restricts SARS-CoV-2 entry into host cells by decreasing ACE2 abundance at the plasma membrane

**DOI:** 10.1186/s13578-024-01331-4

**Published:** 2024-12-20

**Authors:** Piergiorgio La Rosa, Jessica Tiberi, Enrico Palermo, Roberta Stefanelli, Sofia Maria Luigia Tiano, Sonia Canterini, Mirko Cortese, John Hiscott, Maria Teresa Fiorenza

**Affiliations:** 1 Division of Neuroscience, Dept. of Psychology, University La Sapienza, Via dei Sardi 70, 00185 Rome, Italy; 2 European Center for Brain Research, IRCCS Fondazione Santa Lucia, Via del Fosso di Fiorano 64, 00143 Rome, Italy; 3 PhD Program in Behavioral Neuroscience, Sapienza University of Rome, Rome, Italy; 4 Istituto Pasteur Italia-Cenci Bolognetti Foundation, Viale Regina Elena 291, 00161 Rome, Italy; 5 Telethon Institute of Genetics and Medicine, TIGEM, Via Campi Flegrei, 34, 80078 Pozzuoli, Italy; 6 Universitá della Campania Luigi Vanvitelli, Via Vivaldi, 43, 81100 Caserta, Italy

**Keywords:** Spike-ACE2 interaction, ACE2, Virus entry, Lipid dyshomeostasis

## Abstract

**Background:**

The Niemann Pick C1 (NPC1) protein is an intracellular cholesterol transporter located in the late endosome/lysosome (LE/Ly) that is involved in the mobilization of endocytosed cholesterol. Loss-of-function mutations in the *NPC1* gene lead to the accumulation of cholesterol and sphingolipids in LE/Ly, resulting in severe fatal NPC1 disease. Cellular alterations associated with NPC1 inactivation affect both the integrity of lipid rafts and the endocytic pathway. Because the angiotensin-converting enzyme 2 (ACE2) and type 2 serine transmembrane protease (TMPRSS2), interactors of the SARS-CoV-2 Spike protein also localize to lipid rafts, we sought to investigate the hypothesis that NPC1 inactivation would generate an intrinsically unfavorable barrier to SARS-CoV-2 entry.

**Results:**

In this study, we show that inhibition of the cholesterol transporter activity of NPC1 in cells that express both ACE2 and TMPRSS2, considerably reduces SARS-CoV-2 infectivity, evaluated as early as 4 h post-infection. Mechanistically, treatment with NPC1 specific inhibitor U18666A relocalizes ACE2 from the plasma membrane to the autophagosomal/lysosomal compartment, thereby reducing SARS-CoV-2 entry into treated cells. Reduction of viral entry was observed for both fully infectious SARS-CoV-2 virus and with a pseudotyped VSV-Spike-GFP virus. For instance, U18666A-treated Caco-2 cells infected with the pseudotyped VSV-Spike-GFP showed a > threefold and > 40-fold reduction in virus titer when infectivity was measured at 4 h or 24 h post-infection, respectively. A similar effect was observed in CRISP/R-Cas9-edited Caco-2 cells, which were even more resistant to SARS-CoV-2 infection as indicated by a 97% reduction of viral titers.

**Conclusion:**

Overall, this study provides compelling evidence that the inhibition of NPC1 cholesterol transporter activity generates a cellular environment that hinders SARS-CoV-2 entry. ACE2 depletion from the plasma membrane appears to play a major role as limiting factor for viral entry.

**Supplementary Information:**

The online version contains supplementary material available at 10.1186/s13578-024-01331-4.

## Introduction

Cholesterol that is taken up through the endocytic pathway relies on the activity of the intracellular cholesterol transporter Niemann-Pick C1 (NPC1), which delivers cholesterol to various cellular compartments [1–3]. NPC1 is a 13 transmembrane domain protein of the late endosome/lysosome (LE/Ly) limiting membrane involved in the egress of endocytosed cholesterol into the cytosol [3]. Loss-of-function mutations in the *NPC1* gene cause endocytosed unesterified cholesterol and other sphingolipids to clog the LE/Ly compartment [4]. The impairment of endocytic sorting/degradation/recycling is responsible for a severe and ultimately fatal inherited disease with systemic involvement, called Niemann-Pick type C (NPC) disease [5–7].

Because of entrapment within LE/Ly, normal amounts of cholesterol and sphingolipids fail to reach the plasma membrane (PM) or the endoplasmic reticulum (ER), where cholesterol homeostasis is fundamental to various signaling mechanisms [8, 9]. For instance, at the level of the PM, cholesterol is dynamically partitioned between the two fractions of accessible cholesterol and phospholipid (mainly sphingomyelin)-complexed cholesterol. Accessible cholesterol is metabolically active and is readily used for covalent modification of cholesterol-regulated proteins [10], whereas sphingolipid-complexed cholesterol generates plasma membrane dynamic platform named “rafts”. Here, cholesterol moieties ensure tight packaging of the fatty acid chains of sphingolipids, generating regions with high structural order to which various proteins are targeted [11, 12].

The integrity of lipid rafts and the endocytic pathway are crucial for the initial steps of enveloped virus infection [13–16]. The lipid envelope of SARS-CoV-2 is coated with the homotrimeric Spike (S) glycoprotein that mediates viral particle docking to the host cell receptor angiotensin-converting enzyme 2 (ACE2) [17–19]. After binding to ACE2, the S protein is cleaved by the type 2 serine transmembrane protease TMPRSS2, a membrane-bound enzyme, to mediate viral/plasma membrane fusion [20]. Specifically, the S1 subunit of the S protein on the virion surface harbors the receptor binding domain (RBD), while S2 is anchored to the virion membrane and contains the fusion peptide. The RBD-ACE2 binding induces conformational changes in the S protein (referred to as “priming”), as well as in ACE2, exposing S2 to TMPRSS2 cleavage. This provides a crucial prerequisite for the fusion between viral and cell membranes and nucleocapsid entry into the cytoplasm of host cell [20, 21]. Alternatively, the S protein can undergo a series of cleavages by endolysosomal cathepsin B and L proteases when the virus is internalized via endocytosis [18, 22–24]. Based on the requirement of S proteolysis for host cell entry, there are two main routes for viral entry: (i) the fusion of viral and cellular membranes may occur at the host cell plasma membrane or (ii) following endosomal uptake. Which route prevails largely depends on TMPRSS2 expression [23, 25, 26]. In cells that do not express TMPRSS2, S proteolysis can only take place in late endosome/lysosomes where cathepsins localize [23], whereas in cells that do express TMPRSS2 both fusion at the cell surface or at the early endosomal membranes may occur [23]. Besides TMPRSS2, pH has been shown to be a critical determinant of viral entry mechanisms into host cells [27].

It has been shown that the enveloped Ebola virus requires NPC1 as an essential endosomal/lysosomal entry receptor [28, 29]. Additionally, a direct involvement of NPC1 on SARS-CoV-2 entry has been recently reported, by showing that NPC1 binds the RBD of S protein when SARS-CoV-2 is internalized via the endocytic pathway [30].

Apart from the direct involvement of NPC1 in the viral entry as documented by the aforementioned research, we postulated that protection against SARS-CoV-2 virus infection could be obtained through cholesterol dyshomeostasis caused by the inhibition of NPC1 cholesterol transporter activity. Specifically, we postulated that the reduction in plasma membrane (PM) cholesterol achieved by cell treatment with U18666A [31] would provide protection against SARS-CoV-2 entry. This is since cholesterol depletion modifies the biochemical and biophysical properties of PM [32], whereas the S protein-ACE2 interaction and the endocytic pathway [33] depend on the correct arrangement of microdomains rich in cholesterol [13]. To ascertain our hypothesis, we exploited cells from organs that are highly susceptible to SARS-CoV-2 infection and that express both ACE2 and TMPRSS2, including human intestine (Caco-2) and lung (Calu-3) cells and monkey kidney cells expressing human TMPRSS2 (VERO-76). NPC1 inactivation was accomplished either by using the drug U18666A or by CRISPR/R-Cas9 *NPC1* gene editing, and cell infection was carried out using a pseudotyped vesicular stomatitis virus (VSV) expressing the SARS-CoV-2 S protein [34]. Our findings demonstrate that NPC1 inactivation interferes with ACE2 levels on the plasma membrane, thereby reducing SARS-CoV-2 entry. These observations were confirmed both during infection with SARS-CoV-2 virus and with S-pseudotyped VSV. Overall, these findings demonstrate that interference with NPC1 activity and cholesterol metabolism may represent a potential target for inhibiting SARS-CoV-2 infectivity.

## Materials and methods

### Cell culture and treatments

African green monkey kidney Vero-E6 and VERO-76 (hTMPRSS2) cells (RRID: CVCL_0603), the intestinal epithelial cell line Caco-2 (RRID: CVCL_0025), and human airway epithelial Calu-3 cells (RRID: CVCL_0609) were cultured in Dulbecco’s modified Eagle’s medium (DMEM, Merck, cat #D5671) supplemented with 10% fetal bovine serum (FBS, Merck, cat #F2442), 100 U/mL penicillin/streptomycin (Merck, cat #P4333), sodium pyruvate (Merck, cat #S8636), MEM nonessential amino acid (Merck, cat #M7145) and Normocin (InvivoGen, cat #ant-nr1) in a humidified incubator with 5% CO_2_ at 37 °C. The NPC1 inhibitor U18666A (cat # U3633) and methyl-β-cyclodextrin (MβCD, cat # C4555) were purchased from Merck. U18666A was dissolved in DMSO to obtain a 2 mM stock solution; MβCD was dissolved in culture medium at 2 mM concentration.

### Filipin staining and immunofluorescence

The cells were fixed with 4% (v/v) PFA (Merck, cat #F8775) for 15 min and washed three times with Dulbecco’s phosphate-buffered saline (D-PBS, Euroclone, cat #ECB4004L). For filipin staining, cells were incubated with filipin complex (Merck, cat #F-9765; 0.15 mg/mL in D-PBS) for 2 h, and after several washes in D-PBS, the cells were visualized by fluorescence microscopy. For immunofluorescence assays, fixed cells were permeabilized with 0.1% Triton X-100 (Merck, cat #T8787) in D-PBS supplemented with 1% BSA (Merck, cat #A2153) and incubated overnight at 4 °C with the following primary antibodies: α-NPC1 (1:100, cat #ab134113, Abcam), α-ACE2 (1:200, cat #66699-1-Ig, Proteintech), α-ACE2 (1:100, cat MA5-32307, Thermo Fisher), α-TMPRSS2 (1:200, cat #14437-1-AP, Proteintech), α-SPIKE (1:100, cat#703958, Invitrogen), α-LC3-B (1:100, cat #GTX632501, GeneTex), and α-LAMP2 (1:100, cat #MA1-205, Thermo Fisher). The secondary antibodies used were Alexa Fluor 488-conjugated anti-rabbit IgG (Invitrogen, cat #A11070) and Alexa Fluor-conjugated anti-mouse IgG (Invitrogen, cat #A32727), both of which were used at a 1:500 dilution for a 1 h incubation at RT. Nuclear staining was performed with Hoechst (Merck, cat #94403) for 15 min. Slides were mounted using Prolong Gold mounting solution (Invitrogen, cat #00-4958-02). Randomly selected fields for each sample were acquired using a DFC3000 G inverted microscope (Leica Geosystems).

### Protein analysis

Cells were lysed in buffer containing 50 mM Tris–HCl (pH 7.6), 150 mM NaCl, 2 mM EDTA, 1% NP-40, and 0.5% Triton X-100 supplemented with protease inhibitor cocktail (SERVA, cat #39102.01). The purpose of using both detergents was to improve the extraction of membrane proteins as compared to the buffer used for total protein lysates that only contains Triton X-100. The isolation of plasma membrane proteins was performed using Minute™ Plasma Membrane-Derived Lipid Raft Isolation kit - cat #LR-042 (Invent Biotechnologies) following manufacturer’s instructions. The fraction enriched of plasma membrane proteins was identified by using suitable markers, including caveolin-1, Na^+^/K^+^ ATPase and ACE2 (Supplementary Fig. 1). The decrease in the abundance of Na^+^/K^+^ ATPase in U18666A-treated cells is consistent with a previous report [35]. After protein separation by SDS‒PAGE and transfer to polyvinyl difluoride (PVDF) membranes (Amersham, United Kingdom), the blots were incubated overnight at 4 °C with the following antibodies: α-ACE2 (1:1000), α-TMPRSS2 (1:2000), α-NPC1 (1:2000), and α-GAPDH (1:5000, cat #E-AB-20059, Elabscience), α-beta-Tubulin (1:5000, cat# E-AB-20043, Elabscience), α-Na^+^/K^+^ ATPase (1:1000, cat# GTX113390, GeneTex), α-Caveolin-1 (1:1000, cat# AMab2910, Abcam) in TBS containing 0.1% Tween-20 and 5% BSA. Protein bands were detected by enhanced chemiluminescence (Advansta, cat #K-12043-D10) and visualized using an iBright 1500 Imaging System (Invitrogen, cat #A43678). The quantification for digitally acquired images was performed using the ImageJ NIH Software.

### RNA isolation and qRT-PCR

Total RNA was extracted using a Total RNA Purification Plus Kit (Norgen Biotek Corp., cat #48400) acc. to manufacturer’s instructions. One microgram of RNA was reverse transcribed using a OneScript Plus cDNA Synthesis Kit (abm, cat #G236). cDNAs were amplified in qPCR assays using 2× SensiFAST SYBR Lo-ROX Mix (Bioline, cat #BIO-94002) following the manufacturers’ instructions. All primers used in the RT‒PCR and qPCR experiments are listed in Supplementary Table 1. Ribosomal protein L34 (RPL34) mRNA was used for normalization in qPCR experiments.

### NPC1 gene editing

Caco-2 cells were co-transfected with eSpCas9-GFP (Merck, cat #ECAS9GFPPR) and sygRNA (Merck, cat #HSPD0000028649) targeting the 4th exon of the *NPC1* gene using Lipofectamine 2000 (Invitrogen, cat #11668-030) according to the manufacturer’s instructions. Cells were harvested 24 h after transfection, and the top EGFP-expressing cells were sorted into 96-well plates as single clones and as enriched EGFP-expressing populations (100 EGFP-positive cells per well) depending on the experiment. To screen for clones with *NPC1* gene disruption, genomic DNA was analyzed by PCR. NPC1 protein depletion and *NPC1* gene disruption were confirmed by Western blotting and qPCR.

### Pseudoviral particle titration and cell infection

VSV-Spike-GFP, a replication-competent VSV expressing eGFP in the first position of the genome as well as a modified version of the SARS-CoV-2 Spike in place of the native VSV glycoprotein, was a kind gift from Prof. David Olagnier (Aarhus University, Aarhus C 8000, Denmark) and was originally obtained from Prof. Paul W. Rothlauf as described [34]. VSV-Spike-GFP was propagated in VERO-76-hTMPRSS2 cells; briefly, the cells were infected with VSV-Spike-GFP at a MOI of 0.01 for 48 h, and the supernatant was collected, centrifuged 5′ at 300×*g* and then filtered through a 0.22 μm bottle-top vacuum filter. The virus was concentrated by ultracentrifugation at 18,000×*g* for 90 min at 4 °C and then purified on a 20% sucrose cushion at 135,000×*g* for 90 min at 4 °C using a Beckman SW32Ti Swing Bucket Rotor. The purified virus was resuspended in PBS, and the viral titer was quantified by flow cytometry analysis of GFP expression in Vero E6 cells (% GFP-positive cells*#infected cells/mL of virus), as previously described [36]. Titers are expressed as IU/mL. In infection experiments, cells were pretreated with U18666A inhibitor or DMSO (vehicle, Ctrl) for 24 h and then incubated with virus (MOI of 0.1) in serum-free medium for 1 h in a humidified incubator with 5% CO_2_ at 37 °C; after incubation, the medium containing the virus was removed, and replaced with fresh complete medium for 24 h prior to FACS or fluorescence microscopy analysis. In a distinct series of experiments WT Caco-2 cells, mock or U18666A-treated, along with NPC1-edited Caco-2 cells were processed similarly, with the exception that they underwent FACS analysis 4 h post-infection.

### Plaque assay

To compute the number of PFU/mL in the supernatants collected from infected Caco-2 clones, 2.5 × 10^5^ Vero E6 cells were seeded in 24-well plates. The day after seeding, tenfold dilutions of the supernatants were added to the Vero E6 cells, and infected cells were incubated at 37 °C for 2 h. After that, the inoculum was removed, and the medium was replaced with 1 mL of 0.9% carboxymethyl-cellulose plaquing medium (Sigma-Aldrich) in MEM. After 72 h of incubation, Vero E6 cells were fixed in 5% formaldehyde for 30 min and subsequently transferred to a 6% formaldehyde bath for 30 min for inactivation before being transported outside the BSL-3 area. The plates were then rinsed with tap water and incubated for 15 min with 1% crystal violet (Sigma-Aldrich) and rinsed again, and the PFU/mL were counted on stained monolayers.

### SARS-CoV-2 infection of Caco-2 clones

The day before infection, 5 × 10^4^ cells of each Caco-2 clone were seeded in a 24-well plate. Twenty-four hours after seeding, the cells were infected in technical duplicates with SARS-CoV-2 (BAVPAT-1) at MOIs of 1 and 0.1. After 2 h, the viral inoculum was removed, the cells were washed twice in PBS, and DMEM supplemented with 2% FBS was added on top. The supernatants were collected at 24 h post infection, and the viral titer was assessed via plaque assay.

For the entry assay, Caco-2 clones were seeded at a confluency of 5 × 10^4^ in 24 well format. The day after seeding, cells were inoculated with SARS-CoV-2 at MOI 50 for 1 h at 4 °C. After synchronization, the viral inoculum was removed, cells were washed twice in cold PBS and incubated with pre-heated DMEM 2% FBS for 4 h at 37 °C. Cells were then washed twice in PBS and lysed for RNA extraction. Camostat Mesylate (Sigma-Aldrich, SML0057) 100 µM was used as positive control (not shown).

### SARS-CoV-2 infection in the presence of a U18666A inhibitor

WT Caco-2 cells were seeded in a 24-well plate at 5 × 10^4^ cells per well, and after 24 h, the cells were treated with either DMSO or 2 µM U18666A. After 24 h of treatment, the medium was replaced with DMEM supplemented with 2% FBS without compound, and the cells were infected with SARS-CoV-2 at a MOI of 0.1. Viral inoculi were removed after 2 h, the cells were washed twice in PBS, and DMEM supplemented with 2% FBS was added to each well. The supernatants were collected at 24 h postinfection, and the viral titers were quantified by plaque assays on Vero E6 cells.

## Results

### Inactivation of NPC1 perturbs ACE2 and TMPRSS2 expression and localization

**Fig. 1:**
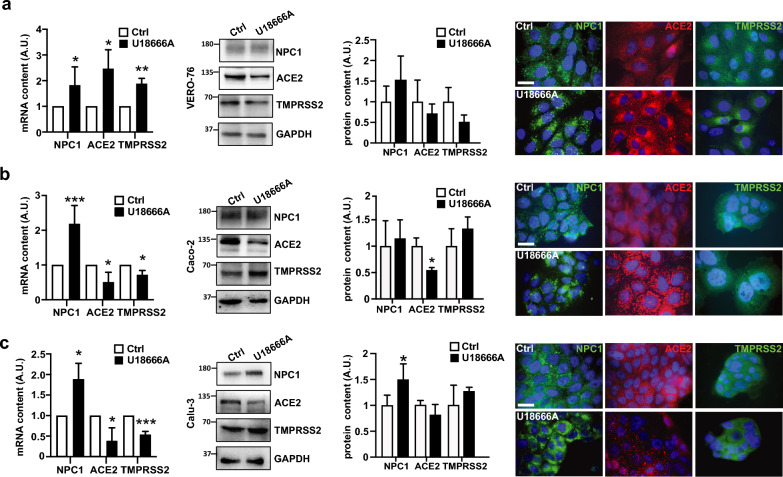
NPC1, ACE2 and TMPRSS2 expression patterns in untreated and U18666A-treated VERO-76, Caco-2 and Calu-3 cells. **a**–**c**, left: Cells were treated with 2 µM U18666A or DMSO (vehicle, Ctrl) for 24 h. Total RNA and proteins were extracted and analyzed by qRT-PCR and Western blotting, respectively. Bars indicate the relative abundance of NPC1, ACE2 and TMPRSS2 transcript levels normalized to RPL34 ribosomal protein RNA and expressed as fold increase compared to control. The data are presented as the means ± SDs of three independent experiments. *p ≤ 0.05, **p ≤ 0.01, ***p ≤ 0.001 vs Ctrl, calculated by Student’s t test. **a**–**c**, center: Representative immunoblots showing the amount of the NPC1, ACE2 and TMPRSS2 proteins, and quantitative densitometry analysis of protein bands normalized to GAPDH. Histograms represent the means ± SDs from three independent experiments. **a**–**c**, right: Representative images of immunofluorescence analysis of VERO-76 cells, Caco-2 and Calu-3 cells treated with 2 µM U18666A or DMSO (vehicle, Ctrl). Nuclei were stained with Hoechst 33342. Images are representative of at least three independent experiments. Scale bar: 50 µM

To evaluate the effect of NPC1 inactivation on SARS-CoV-2 infection, we used the pharmacological inhibitor U18666A, which blocks the cholesterol transport activity of NPC1 [37]. The appropriate U18666A concentration and duration of NPC1 inhibition were determined by dose‒response curve analysis (Supplementary Fig. 2a). The three cell types responded similarly to 24, 48 and 72 h of treatment with 0.02–200 µM U18666A (Supplementary Fig. 2a, b). However, filipin staining revealed that 24 h of treatment with 2 µM U18666A produced a robust NPC1 phenotype with minimal effect on cell survival (Supplementary Fig. 2a, graphs). This observation prompted us to evaluate the mRNA and protein expression of NPC1, ACE2, and TMPRSS2 in VERO-76, Caco-2 and Calu-3 cells [38, 39] that were either untreated or treated for 24 h with 2 µM U18666A (Fig. 1). Drug treatment modified the transcript levels, as shown by a twofold increase in the *NPC1* transcript content in the three cell types. The VERO-76 cells showed a similar increase in ACE2 and TMPRSS2, but the Caco-2 and Calu-3 cells showed approximately 2.5- and 1.5-fold decreases in ACE2 and TMPRSS2, respectively (Fig. 1a–c). The presence of U18666A significantly increased the expression of the NPC1 protein in Calu-3 cells and significantly decreased the expression of ACE2 in Caco-2 cells. VERO-76 and Calu-3 cells showed a moderate decrease in ACE2 abundance (Fig. 1a, b, e, c).

Next, we examined the effect of U18666A treatment on the intracellular localization of NPC1 using immunofluorescence analysis. NPC1 exhibited prominent localization within the perinuclear organelles of all three cell types (Fig. 1a–c, immunofluorescence) [40], while a similar accumulation of ACE2 in the perinuclear region was observed in VERO-76 and Caco-2 cells, but less so in Calu-3 cells. Similarly, VERO-76 showed a TMPRSS2 relocation pattern similar to that of ACE, but TMPRSS2 localization did not change in Caco-2 and Calu-3 cells (Fig. 1a–c). This observation confirms that drug treatment causes the re-localization of NPC1 within perinuclear organelles [40–43] and suggest that concurrent depletion of ACE2 from the plasma membrane may occur while this protein accumulates intracellularly.

### Treatment with U18666A targets NPC1 and ACE2 to the autophagolysosomal compartment

**Fig. 2:**
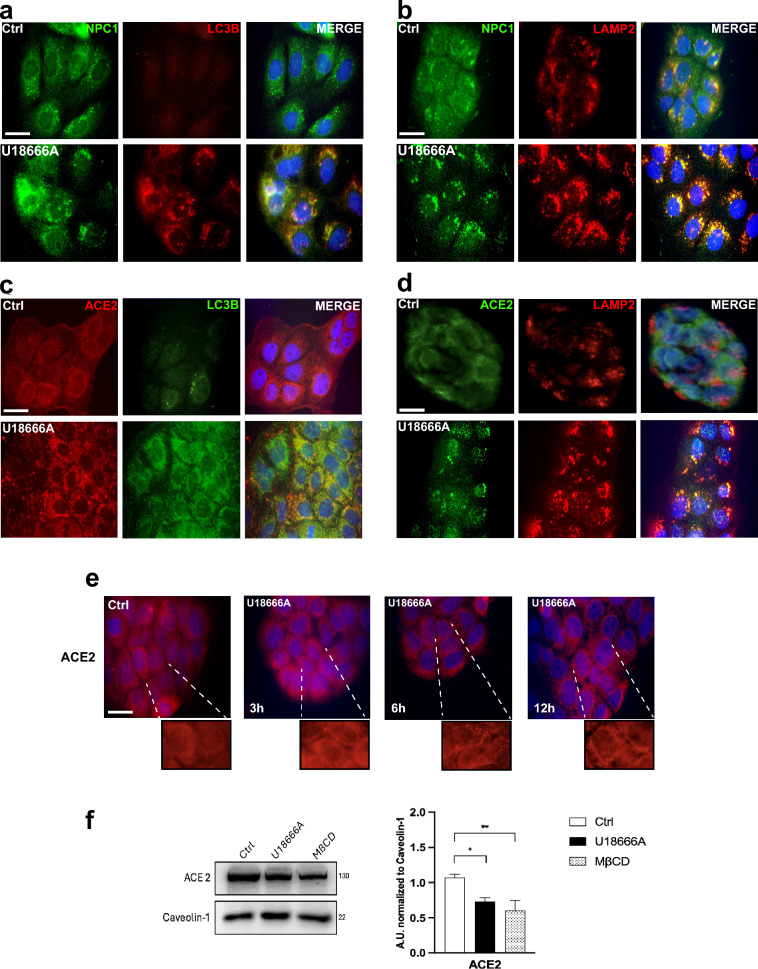
Characterizing how treatment with U18666A affects the intracellular localization of NPC1 and ACE2. **a**–**d** Representative images of immunofluorescence analysis of Caco-2 cells treated with 2 µM U18666A or DMSO (vehicle, Ctrl) for 24 h and then subjected to double immunofluorescence experiments by incubation with the following pairs of antibodies: α-NPC1 (green), α-LC3B (red) (**a**), α-NPC1 (green), and α-LAMP2 (red) (**b**); α-ACE2 (red), α-LC3B (green) (**c**), α-ACE2 (green), and α-LC3B (red) (**d**). Nuclei were stained with Hoechst 33342. Images are representative of at least three independent experiments. Scale bar: 50 µM. **e** Time course immunofluorescence analysis of ACE2 within 0–12 h time frame. **f** Representative immunoblots showing ACE2 and Caveolin-1 abundance in the plasma membrane fraction of control and either U18666A- or MβCD-treated Caco-2 cells, and quantitative densitometry analysis of protein bands normalized to Caveolin-1. Histograms represent the means ± SD from three independent experiments. *p ≤ 0.05; **p ≤ 0.01

The observation that ACE2 acquired perinuclear organelle localization similar to that of NPC1 prompted us to characterize the compartment to which the two proteins were located, with particular reference to endocytic and/or autophagic compartments [31, 44]. Control and U18666A-treated Caco-2 cells were processed by double immunofluorescence analysis using antibodies directed against LAMP-2 and LC3B to detect the lysosomal and autophagosomal compartments, respectively [45]. In treated cells, we observed strong colocalization of NPC1-LC3B and NPC1-LAMP-2 within perinuclear puncta (Fig. 2a, b), which is consistent with the targeting of NPC1 to the autophagosomal/lysosomal compartment. Remarkably, ACE2 was found to substantially colocalize with both LC3B and LAMP-2 (Fig. 2c, d), as well as with NPC1 (Supplementary Fig. 3a). VERO-76 cells treated with U18666A showed a similar pattern of NPC1 and ACE2 targeting to the autophagosomal/lysosomal compartment (Supplementary Fig. 3b–e).

This observation prompted us to determine the temporal dynamics of ACE intracellular re-localization by immunofluorescence analysis of U18666A-treated Caco-2 cells 3, 6 and 12 h following exposure to the drug. We found that the perinuclear ACE2 accumulation progressively increased over the time (Fig. 2e) and was accompanied by a significant decrease of ACE2 content at the plasma membrane (Fig. 2f). A similar decrease of ACE2 content at the plasma membrane was observed when Caco-2 cells were treated with methyl-β-cyclodextrin MβCD, as a means to achieve plasma membrane cholesterol depletion [4]. We therefore conclude that NPC1 inactivation disrupts ACE2 localization at the plasma membrane by altering cholesterol at the plasma membrane.

### NPC1 inhibition confers resistance to SARS-CoV-2 Spike-pseudotyped virus entry and propagation

**Fig. 3:**
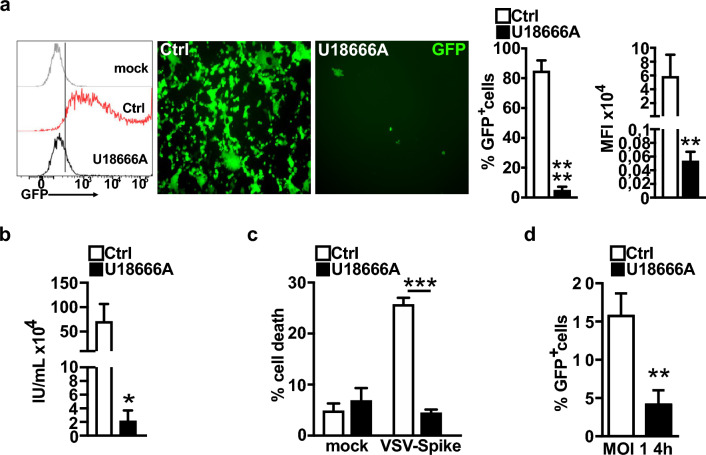
The inhibition of NPC1 counteracts VSV-Spike entry. **a** Cells were seeded in a 12-well plate and treated with either 2 µM U18666A inhibitor or DMSO (vehicle, Ctrl) for 24 h. The cells were then infected with VSV-Spike-GFP (MOI of 0.1), harvested 24 h after infection and analyzed for GFP expression. An example of a FACS analysis output showing the percentage of GFP-positive cells/experimental/group. Representative images of immunofluorescence analysis of VSV-Spike-GFP-infected cells and quantitative analysis of GFP-positive cells (histograms). **p ≤ 0.01; ****p ≤ 0.0001. **b** Control and U18666A-treated cells were infected with VSV-Spike-GFP (MOI of 0.1). The supernatants were collected after 24 h and used to infect Vero E6 cells; at 7 h postinfection, the fraction of GFP-positive cells was quantified by flow cytometry, and the viral titer, expressed as infection units (IU), was determined. Histograms represent the mean ± SD from three independent experiments. *p ≤ 0.05. **c** Cells were harvested 24 h after VSV-Spike-GFP (MOI of 0.1) infection and stained with 7-AAD to quantify the fraction of dead cells by flow cytometry. Histograms represent the mean ± SD from three independent experiments. ***p ≤ 0.001. **d** Caco-2 cells were seeded in a 24-well plate and where either mock-(DMSO) or U18666A-treated (2 µM). After 24 h cells were infected with VSV-Spike-GFP (MOI 1) and the fraction of GFP-positive cells was determined by flow cytometry at 4 h post-infection. **p ≤ 0.01

To directly investigate the possibility that ACE2 depletion at the plasma membrane hindered the docking of the SARS-CoV-2 Spike protein to the plasma membrane of host cells, U18666A-treated or mock Caco-2 cells were infected with pseudotyped VSV-Spike-GFP virus [34], and infectivity was measured by GFP expression. Treatment with U18666A significantly decreased GFP fluorescence by more than 90%, demonstrating a very strong inhibition of VSV-Spike-GFP infection (Fig. 3a). Supernatants collected from infected and treated cells were used to infect naïve VERO-E6 and FACS analysis was used to calculate the percent of GFP-positive cells. Consistent with the results observed during primary infection, re-infection experiments showed a strong reduction in secreted infectivity (Fig. 3b). Moreover, the percentage of apoptotic U18666A-treated Caco-2 cells was comparable to that of control and U18666A-treated mock cells (Fig. 3c), providing additional evidence of resistance to virus infection determined by NPC1 inhibition. In contrast, U18666A-untreated infected cells (Fig. 3c) showed a dramatic VSV-induced cytopathic effect, with approximately 30% apoptotic cell death. A lower concentration of U18666A (0.2 µM, 72 h, see dose‒response curves of Supplementary Fig. 2a) produced results that were comparable (not shown). NPC1 inhibition in Calu-3 and VERO-76 cells produced similar patterns of resistance to VSV-Spike-GFP infection and propagation (Supplementary Fig. 4a–f). The decreased vulnerability to VSV-Spike infection was consistent with the marked decrease of Spike expression in VERO-76 cells treated with U18666A compared to the control (p ≤ 0.0001) (Supplementary Fig. 4g). We then investigated whether NPC1 inactivation hampered the initial phases, specifically the entry phase of the pseudoviral life cycle. This was accomplished by quantifying the fraction of GFP-positive cells 4 h after infection, rather than 24 h later. In our experience, 4 h after infection is the earliest time point at which reliable GFP positiveness can be observed [46]. These experiments demonstrated a very strong reduction of GFP-expressing cells − specifically a ~ fourfold reduction − when U18666A treatment preceded pseudoviral infection, confirming that NPC1 inhibition affects viral entry by reducing ACE2 levels on the plasma membrane.

### NPC1 gene editing restricts SARS-CoV-2 infection

The CRISPR/R-Cas9 gene-edited Caco-2 cells were then used to test the resistance to VSV-Spike-GFP entrance caused by the lack of NPC1 activity. Initially, we identified two clones, in which NPC1 protein content was found to be below detection and barely detected, respectively. Nevertheless, the sequencing of the PCR product amplified from the vicinity of the CRISPR/Cas9 gRNA guide revealed the presence of an extra high MW band in one clone, which was caused by a chromosome 19–18 translocation. As such, even though this clone was a straightforward KO, we had to discard it.

**Fig. 4:**
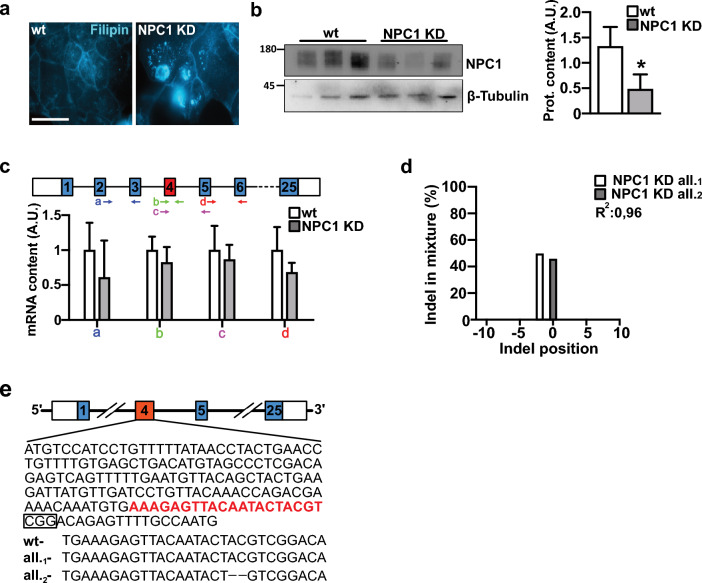
Validation of the genetic inactivation of NPC1 by CRISPR/Cas9. **a** Caco-2 cells were edited using an RNA-guided Cas9 nuclease targeting exon 4, obtaining a clone − NPC1 KD − whose cells closely resemble the typical intracellular cholesterol accumulation of the NPC1 phenotype, as determined by filipin staining. **b** Representative immunoblots and quantitative densitometry analysis of protein bands normalized to beta-Tubulin (bars) showing the presence of less than 50% residual NPC1 protein in NPC1 KD cells compared with that in *WT* cells. **c** A scheme of primer pairs encompassing the CRISPR/Cas9 gRNA-targeted exon 4 nucleotide sequence, which were used to assess transcript amplification. Bars indicate the relative abundance of RT-PCR products amplified from NPC1 KD cells. **d** Trace decomposition analysis performed by analyzing the sequencing of the genomic region targeted by the gRNA used to edit *NPC1*. The graph shows a 2-nucleotide deletion present on one allele (white bar), while the second allele (gray bar) does not exhibit any deletion or insertion. **e** Diagram showing the structure of the *NPC1* gene, including part of the exon 4 gene sequence. The sequence to which the spacer used for the editing experiment pairs, is highlighted in red, while the circled sequence represents the protospacer adjacent motif (PAM). Below, the WT sequence where the Cas9-mediated enzymatic cut occurs, and the sequences of the two *NPC1* coding alleles present in the genome of the NPC1 KD clone. The data are presented as the means ± SDs of three independent experiments. *p ≤ 0.05

Cells of the second clone exhibited intracellular cholesterol accumulation characteristic of NPC1-deficient cells upon filipin staining (Fig. 4a). However, further characterization showed the presence of less than 50% residual NPC1 when analyzed by Western blot assays (Fig. 4b) and a certain degree of immunoreactivity in immunofluorescence assays (Fig. 6c). When the synthesis of *NPC1* transcripts was determined, a slight decrease of *NPC1* transcript amplification was noted with primer pairs either upstream or downstream the targeted exon 4 compared to the control cells.

Consistently, trace decomposition analysis of the output of three distinct rounds of DNA sequencing with Tracking of Indels by Decomposition (TIDE) [47] and the Synthego Performance Analysis, Inference of CRISPR Edits (ICE) v3.0 [48] software revealed that mutagenesis had actually occurred at the expected position in one of the two alleles only (Fig. 4d, e). Therefore, we concluded that this was a knockdown clone (Fig. 4b).

**Fig. 5:**
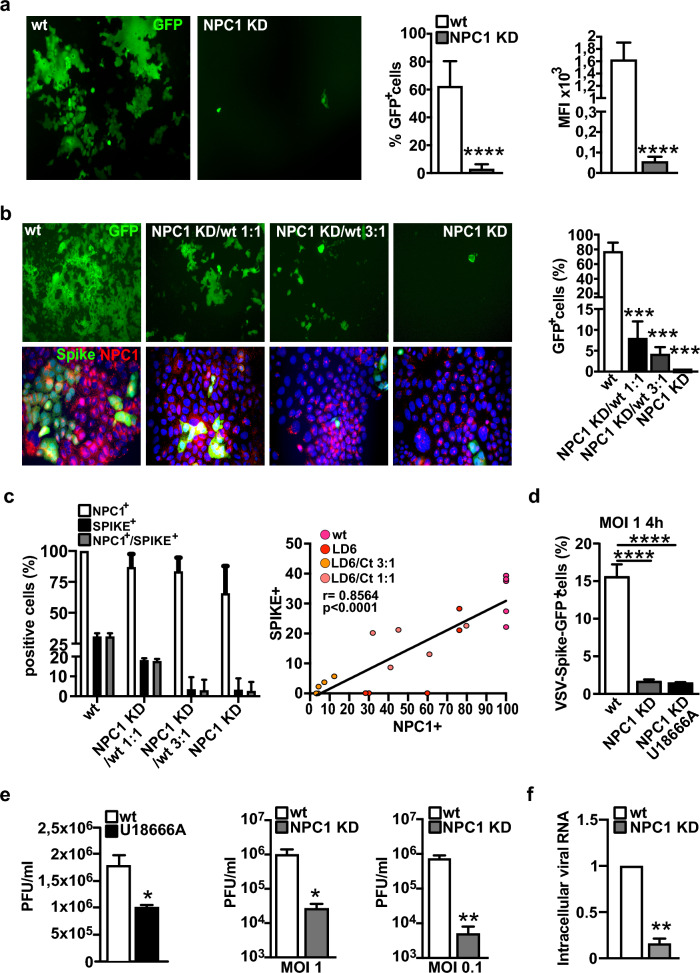
NPC1 knockdown counteracts VSV-Spike-GFP and SARS-CoV-2 infection. **a** Either Caco-2 wild-type (WT) or NPC1 KD, were infected with VSV-Spike-GFP (MOI of 0.1), and pseudoviral infectivity was measured 24 h post infection by determining GFP expression via immunofluorescence and flow cytometry. Histograms represent the mean ± SD from three independent experiments. **b** NPC1 KD clone cells alone or mixed with WT cells at two different ratios, i.e., 1:1 or 3:1, were infected with VSV-Spike-GFP (MOI of 0.1), and pseudoviral infectivity was measured 24 h post infection by determining the fraction of GFP-positive cells (immunofluorescence and bars on the left). The panel below show Spike- and NPC1-expressing cells as detected by double immunofluorescence. **c** NPC1 KD-infected cells were processed for NPC1/Spike double immunofluorescence to determine the relative fraction of NPC1-positive, Spike-positive and NPC1/Spike-positive cells among the total cells. Pearson’s correlation coefficient between NPC1 and Spike expression. **d** Caco-2 WT and NPC1 KD cells were seeded in a 24-well plate and where either mock-(DMSO) or U18666A-treated (2 µM). After 24 h cells were infected with VSV-Spike-GFP (MOI of 1) and the fraction of GFP-positive cells was measured by flow cytometry at 4 h post-infection. **e** Untreated and U18666A-treated Caco-2 cells were infected with native SARS-CoV-2 virus, and released infectivity was determined from supernatants collected 24 h post infection (bars on the left). WT and NPC1 KD cells were infected with SARS-CoV-2 at either 1 or 0.1 MOI, and released infectivity was determined from supernatants collected 24 h post infection (middle and right bars). Histograms represent the mean ± SD from three independent experiments. *p ≤ 0.05; **p ≤ 0.01; ***p ≤ 0.001; ***p ≤ 0. 001. **f** Caco-2 WT and NPC1 KD cells were incubated on ice for 1 h with SARS-CoV-2 (MOI of 50). Cells were washed with cold PBS to remove the inoculum, then warm medium was added before moving the infected cells at 37 °C. After 4 h, cells were collected, intracellular RNA was extracted, and viral genomes were quantified by qRT-PCR. Mean and SD of three independent experiments are shown. Significance was calculated with one-sample t-test. **p ≤ 0.01

In agreement with the partial loss-of-function and the solid NPC1 phenotype NPC1 KD cells were resistant to SARS-CoV-2 infection when challenged with VSV-Spike-GFP. Indeed, the infection of NPC1 KD cells with pseudotyped VSV-Spike-GFP virus resulted in ~ 3% GFP-positive cells compared to ~ 60% GFP-positive cells in WT Caco-2 cells (Fig. 5a). As additional proof of the strict dependency of virus infection on NPC1 expression, we mixed NPC1 KD cells with WT Caco-2 cells at different ratios and observed that the increase in the fraction of NPC1 KD cells was accompanied by a decrease in GFP positivity (Fig. 5b). The relationship between NPC1 expression and virus propagation was further supported by the direct correlation observed between the fraction of Spike- and NPC1-expressing cells within the mixed population (Fig. 5b, c; r 0.8564; p < 0.0001). To ascertain if NPC1 knockdown impeded the entry phase of the pseudoviral life cycle in a manner comparable to what we saw upon NPC1 inactivation (Fig. 3d), we quantified the fraction of GFP-positive cells 4 h after infection, instead of 24 h later. These experiments demonstrated a very strong reduction of the fraction of GFP-positive NPC1 KD cells − specifically a ~ tenfold reduction, as compared to the WT cells. The exposure of NPC1 KD cells to U18666A had no additive effect.

The protective effect of NPC1 inactivation/knockdown against viral infection was further corroborated by infecting untreated and U18666A-treated Caco-2 cells with pathogenic SARS-CoV-2 (BAVPAT-1). SARS-CoV-2 infectivity was 42% lower in U18666A-treated cells than in control cells, as determined by virus titration (Fig. 5d). A dramatic reduction in SARS-CoV-2 infectivity was also observed when WT and NPC1 KD cells were infected with pathogenic SARS-CoV-2, with an observed reduction in secreted infectivity of 97%. Specifically, SARS-CoV-2 infectivity was reduced by 97% at a MOI of 1 and further decreased to 99% at a MOI of 0.1 (Fig. 5d). To test if the reduction in SARS-CoV-2 infectivity was due specifically to an effect on the early steps of viral infectious cycle, we performed a synchronized infection of Caco-2 WT and NPC1 KD cells by incubating the cells with the virus on ice for 1 h. After extensive washes with cold PBS to remove residual inoculum, infection was allowed to proceed for 4 h at 37 °C. The time point was chosen based on earlier studies performed in the same cell lines that demonstrated low level of replication after 4 h post infection [49]. In line with the results observed with the VSV-S pseudotyped virus, a strong decrease in the intracellular viral RNA was observed in NPC1 KD cells.

**Fig. 6:**
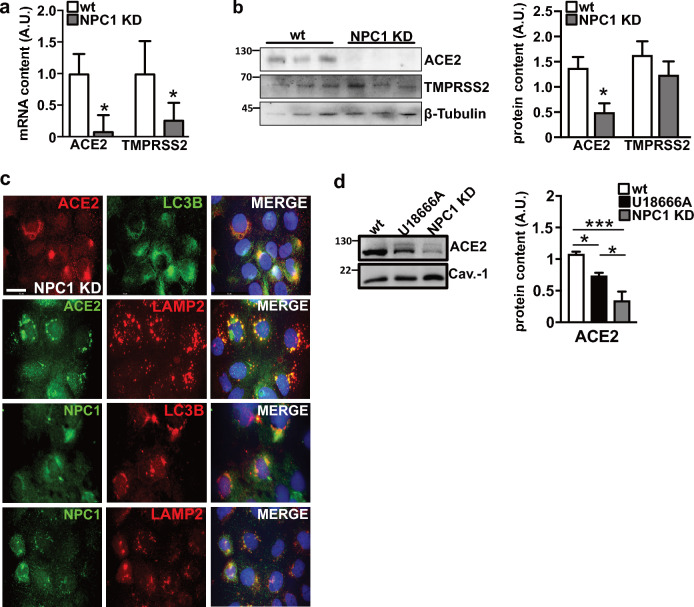
Expression patterns of ACE2 and TMPRSS2 in NPC1 KD cells. Total RNAs and proteins were extracted and analyzed by qRT-PCR and Western blot. **a** Bars indicate relative abundance of ACE2 and TMPRSS2 transcript levels normalized to RPL34 ribosomal protein RNA and expressed as fold-increase over control. **b** Representative immunoblots and quantitative densitometry analysis of protein bands normalized to β-Tubulin. The same β-Tubulin blot of Fig. 4b is displayed here because the three proteins, NPC1, ACE2 and TMPRSS2 were analyzed from the same blot. Data are presented as mean ± SD of three independent experiments. *p ≤ 0.05, **p ≤ 0.01. **c** NPC1 and ACE2 expression patterns. Representative images of double immunofluorescence analyses performed by reacting cells with the following pairs of antibodies: α-ACE2 (red), α-LC3B (green), α-ACE2 (green), and α-LAMP2 (red); α-NPC1 (green), α-LC3B (red), α-NPC1 (green), α-LAMP2 (red); Nuclei were stained with Hoechst 33342. Images are representative of at least three independent experiments. Scale bar: 50 µM. **d** Representative immunoblots showing ACE2 and Caveolin-1 abundance in the plasma membrane fraction of untreated and U18666A-treated Caco-2 cells and NPC1 KD cells, and quantitative densitometry analysis of protein bands normalized to Caveolin-1. Histograms represent the means ± SD from three independent experiments. *p ≤ 0.05; ***p ≤ 0.001

To gain a mechanistic understanding of the resistance of NPC1 KD cells to SARS-CoV-2, we determined the expression patterns of ACE2 and TMPRSS2. ACE2 and TMPRSS2 transcript and protein levels were lower in NPC1 KD cells compared to WT, with ACE2 expression being downregulated more than TMPRSS2 expression. Furthermore, ACE2 intracellular accumulation and colocalization with LC3B and LAMP2 (Fig. 6c) are consistent with the expression patterns observed in cells treated with U18666A. The dependence of SARS-CoV-2 efficient entry on ACE2 expression at the PM is strengthen by the finding that ACE2 abundance at the PM is significantly lower in NPC1 KD cells, compared to WT (Fig. 6d).

## Discussion

The lipid microenvironment of various cellular organelles is known to be a critical factor for the infectivity of enveloped viruses, including SARS-CoV-2 [15, 24, 50, 51]. For instance, the depletion of cholesterol and sphingolipids at the plasma membrane (PM) hinders virus entry by interfering with the docking of the Spike protein to the ACE2 receptor and the subsequent TMPRSS2 priming or clathrin-/caveoale-dependent endocytosis [13, 20, 33]. The disturbance of nonvesicular lipid transport via membrane contact sites (MCVs), with particular reference to LE/L-ER MCVs, hampers virus replication because double membrane vesicles (DMVs), which act as platforms for viral replication, originate from cholesterol-rich ER sub-compartments [52].

The lysosomal cholesterol transport activity of NPC1 guarantees that PM and organelle membranes have an adequate amount of cholesterol and sphingolipids, whereas U18666A—a drug that binds the sterol sensing domain of NPC1 and inhibits its activity, is widely used to model Niemann Pick C disease [37, 53–56] and study how NPC1 inhibition affects viral entry and replication [28, 57–60]. It has been shown that U18666A effectively hampers the ability of many viruses, including SARS-CoV-2, to entry the host cells by inhibiting clathrin-dependent endocytosis [33]. This phenomenon has been associated with decreased cholesterol content at the PM, which modifies cholesterol-rich microdomains necessary for ACE2 localization and endocytic pathways. It is thought that multiple attachment sites for S proteins are provided by the high concentration of ACE2 at these microdomains, which then facilitates S-binding to ACE2 and the initiation of the entry process [16, 33, 61, 62].

We were able to focus this study on the early stages of virus infection, when S-mediated events include virus attachment to the host surface and induction of the fusion with cell membrane, by using a VSV virus, pseudotyped with the SARS-CoV-2 Spike protein in place of the VSV-G, to infect cell types expressing both ACE2 and TMPRSS2 [20, 34].

Similar phenotypes of intracellular cholesterol build up, increased expression of NPC1 mRNA, and NPC1 protein targeting to perinuclear organelles were observed in all cell types in response to U18666A treatment (reviewed in [31]). However, our finding that NPC1 inhibition in the presence of U18666A relocalizes ACE2 from the plasma membrane (PM) to the autophagosomal/lysosomal compartment is new, and underlines a change in ACE2 trafficking and/or partitioning between different cell compartments. In particular, the build-up of ACE2 in the autophagosomal-lysosomal compartment is in line with the defective fusion of autophagosomes and lysosomes caused by U18666A-dependent NPC1 inhibition [63]. The increase in ACE2 intracellular immunoreactivity at 3 h after treatment initiation and its progressive increase during the 3–12 h time frame suggest that NPC1 inhibition has a significant impact on ACE2 localization. We demonstrated that the rise in intracellular ACE2 concentration correlates with a corresponding fall in its abundance at the PM, which is thought to be the result of a depletion of PM cholesterol [13]. This is further confirmed by the similar decrease in PM ACE2 content observed with MβCD treatment. A similar effect on ACE2 expression and trafficking, along with a substantial reduction of the interaction between the S1 ectodomain of Spike and ACE2 has been recently reported in Caco-2 cells treated with fluvastatin and simvastatin—two lipid-lowering compounds [64].

Concerning the ACE2 PM-to-intracellular compartment mobilization, we consider two possible scenarios: either newly synthesized ACE2 protein is prevented from moving to the PM by a malfunctioning ER-Golgi secretory pathway [63], or reduced cholesterol levels at the PM cause ACE2 internalization by upsetting the ideal microenvironment for its activity [50, 65]. The first scenario is supported by the evidence that the loss of function of RAB7A − a small GTPase that is involved in vesicular transport − confers resistance to SARS-Cov-2 infection by reducing ACE2 cell surface expression and increasing its endosomal accumulation [61]. Either way, both scenarios are in line with the fact that the low concentrations of U18666A used in this study impairs the delivery of cholesterol from lysosomes to both the PM and ER [66], thus altering cholesterol rich microdomains of the PM and intracellular vesicular movements.

The lowering of ACE2 abundance at the PM likely explains the high resistance of U18666A-treated cells to SARS-CoV-2 infection. In fact, cholesterol-regulated ACE2 localization to suitable PM lipid clusters promotes viral attachment and entry. Therefore, we can presume that NPC1 inhibition with U18666A hinders SARS-CoV-2 infection by modifying the cholesterol-dependent ACE2 association with the PM lipid microenvironment required for efficient virus attachment to host cells [65]. This conclusion is in line with the study by Takano et al., who showed that membrane cholesterol levels are critical for the successful infection of host cells by feline coronavirus [59]. Additionally, a recent study demonstrated that SARS-CoV-2 infectivity is strictly dependent on the portioning of ACE2 to appropriate PM lipid clusters [67]. Conversely, because lysosomal pH is barely affected by the relatively low concentration of U18666A (2 μM and 0.2 μM) that was used in this study to achieve NPC1 inhibition, the possibility of an antiviral effect based on the blockade of cathepsin protease activity by an increase in lysosomal pH seems unlikely [68].

The recent study by Khan et al. [30] has shown through in silico and biochemical evidence that NPC1 directly binds the RBD of S-protein as ACE2 does. This direct interaction between NPC1 and Spike RBD takes place in late endosomes, where NPC1 localizes, and mediates the fusion between virus and endosomal membranes. However, upstream from this direct action of NPC1, cholesterol dyshomeostasis at the PM, brought on by the inhibition of the cholesterol transport activity of NPC1, has a significant impact on the endocytic pathway itself. Indeed, NPC1 inhibition blocks viral entry trough the endocytic route [33]. By combining the results of Khan’s research with those of previous investigations and current study, we can draw the conclusion that SARS-CoV-2 entry into the host cells is influenced by both the direct interaction between NPC1 and Spike RBD and the cholesterol transporter activity of NPC1. One of the primary differences between the two is that NPC1’s binding to the S-RBD is limited to late endosomes, which is the compartment where NPC1 localizes, whereas fusion mechanisms happening at the PM or in early endosomes are more likely influenced by the cholesterol transporter activity, as suggested by our results. Therefore, it is reasonable to conclude that targeting both direct NPC1 − Spike-RBD interaction and NPC1 cholesterol transporter activity may synergistically limit SARS-CoV-2 entry. 

In addition to the impact on viral entry, our results showed a noteworthy reduction in released infectivity in U18666A-treated cells. Although this is in line with the impairment of viral replication discussed above, the egress of virions from infected cells may also be impaired. This is because SARS-CoV-2 exploits lysosomal trafficking instead of the secretory pathway for egress [69]. Consequently, disruption of lysosomal transport linked to NPC1 inactivation [70] is expected to impact the release of viral particles.

Further validation of the crucial function of NPC1 in controlling cell susceptibility to SARS-CoV-2 infection was achieved by CRISP/R-Cas9 knock down of NPC1 in Caco-2 cells. Although mutagenesis had occurred in only one of the two alleles, these cells, named NPC1 KD, showed significantly greater resistance to infection when challenged with VSV-Spike-GFP under conditions comparable to those used for U18666A-treated cells. These findings were further corroborated by the significant reduction in infection rates observed in either U18666A-treated Caco-2 or NPC1 KD cells challenged with natural SARS-CoV-2. Furthermore, the reduction of ACE2 at the PM and its targeting to the autophagolysosomal compartment in NPC1 KD cells indicate that the molecular basis of infection resistance in NPC1 KD cells and U18666A-treated cells is similar.

Lastly, we also provided a more concrete demonstration that the deficit of NPC1 function has a protective effect against VSV-Spike-GFP infection by infecting increasing amounts of NPC1 KD cells mixed with a fixed amount of *WT* cells. In this context, analysis of Pearson correlation coefficients revealed a significant linear correlation between NPC1-expressing cells and Spike-positive cells, confirming the strong association between NPC1 expression and infection ratios.

## Conclusions

This study outlines the significant convergence between the cellular alterations associated with the inactivation of the intracellular cholesterol transporter activity of NPC1 and the mechanistic processes of SARS-CoV-2 infectivity. Specifically, the study demonstrated that the expression of ACE2 at the plasma membrane is a critical determinant for efficient viral entry.

## Supplementary Information

Supplementary Material 1.

Supplementary Material 2.

Supplementary Material 3.

Supplementary Material 4.

Supplementary Material 5.

Supplementary Material 6.

## Data Availability

The datasets generated during the current study are available from the corresponding author upon reasonable request.
