# Supplementary figures and images for: The inactivation of the Niemann Pick C1 cholesterol transporter restricts SARS-CoV-2 entry into host cells by decreasing ACE2 abundance at the plasma membrane
Source: Cell Biosci. 2024 Dec 20;14:148. doi: 10.1186/s13578-024-01331-4 (PMC11662611; doi:10.1186/s13578-024-01331-4)

# Fig1s

a

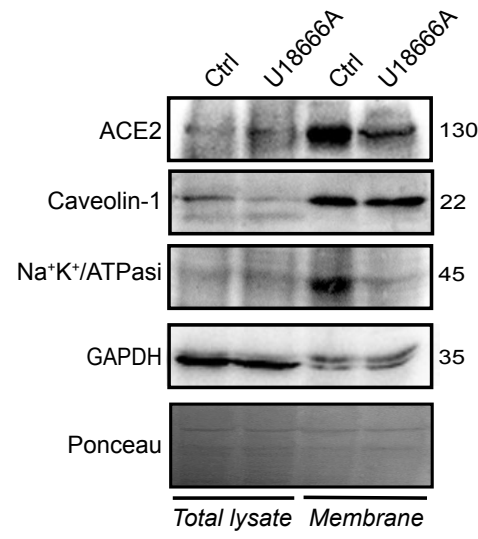

Supplement: Supplementary file 1 — Supplementary Material 1. [file 13578_2024_1331_MOESM1_ESM.pdf]

Fig2s

a

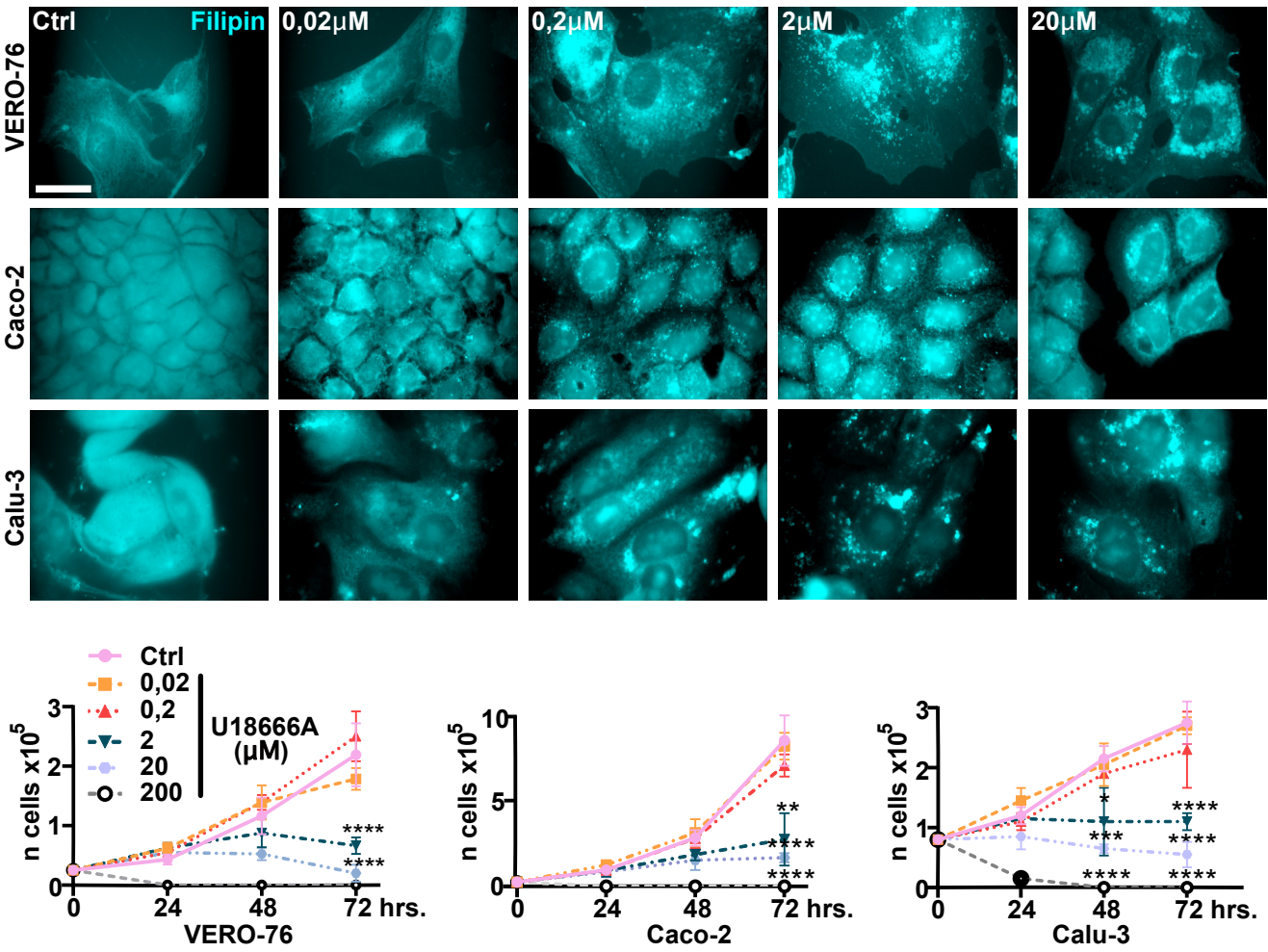

b

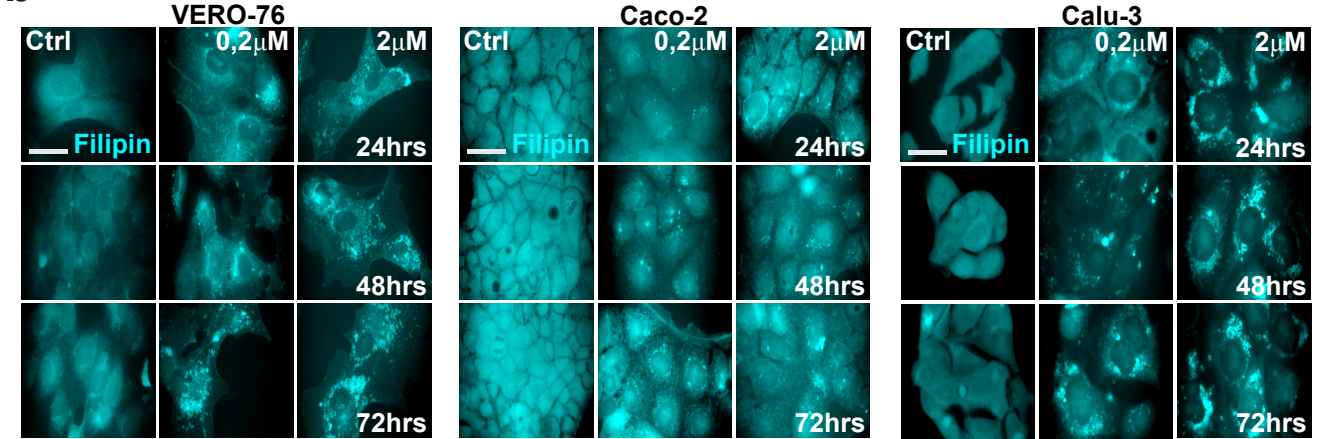

Supplement: Supplementary file 2 — Supplementary Material 2. [file 13578_2024_1331_MOESM2_ESM.pdf]

**Fig3s**

**a**

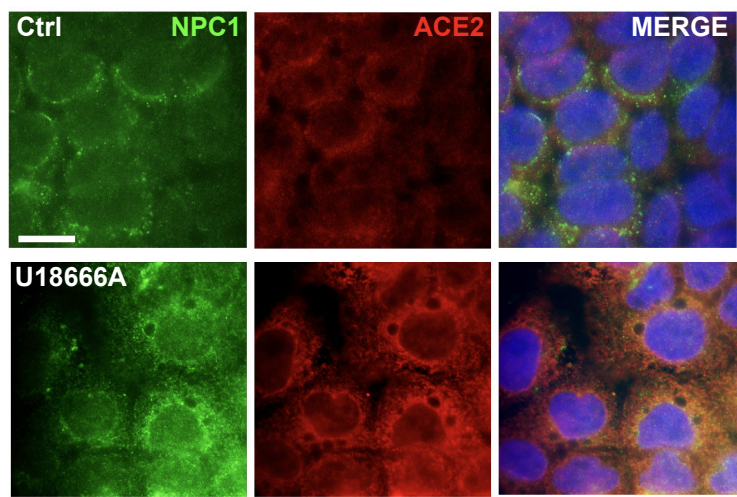

**b**

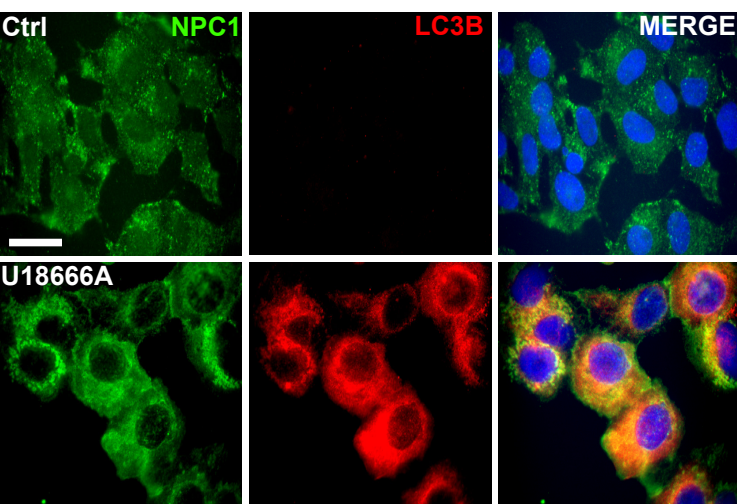

**c**

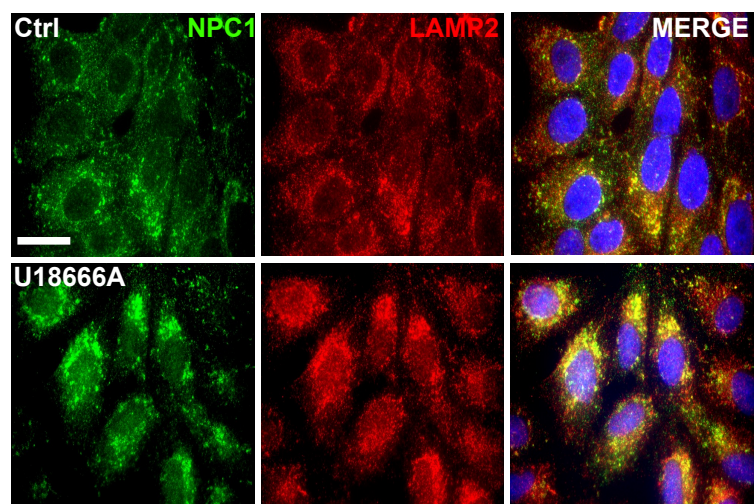

**d**

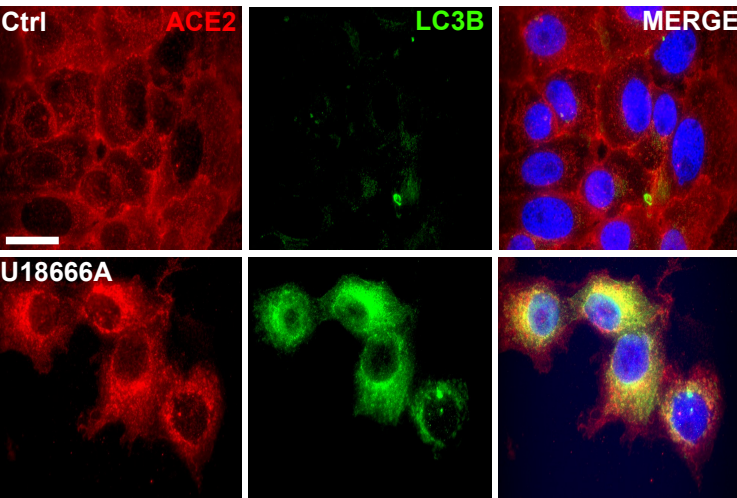

**e**

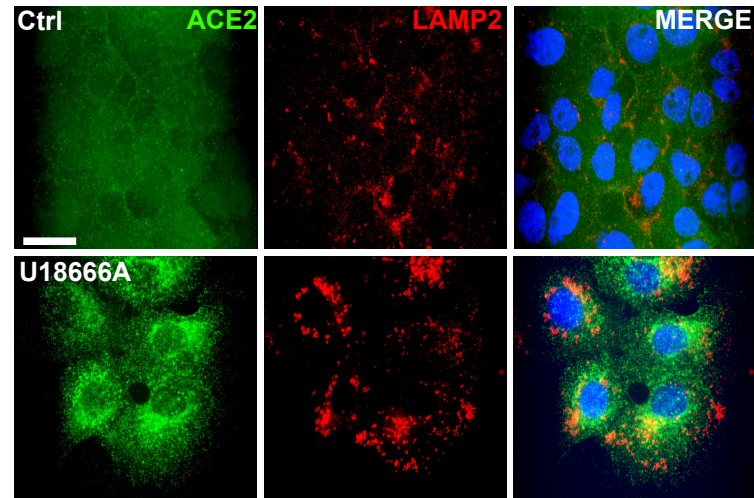

Supplement: Supplementary file 3 — Supplementary Material 3. [file 13578_2024_1331_MOESM3_ESM.pdf]

**Fig4s**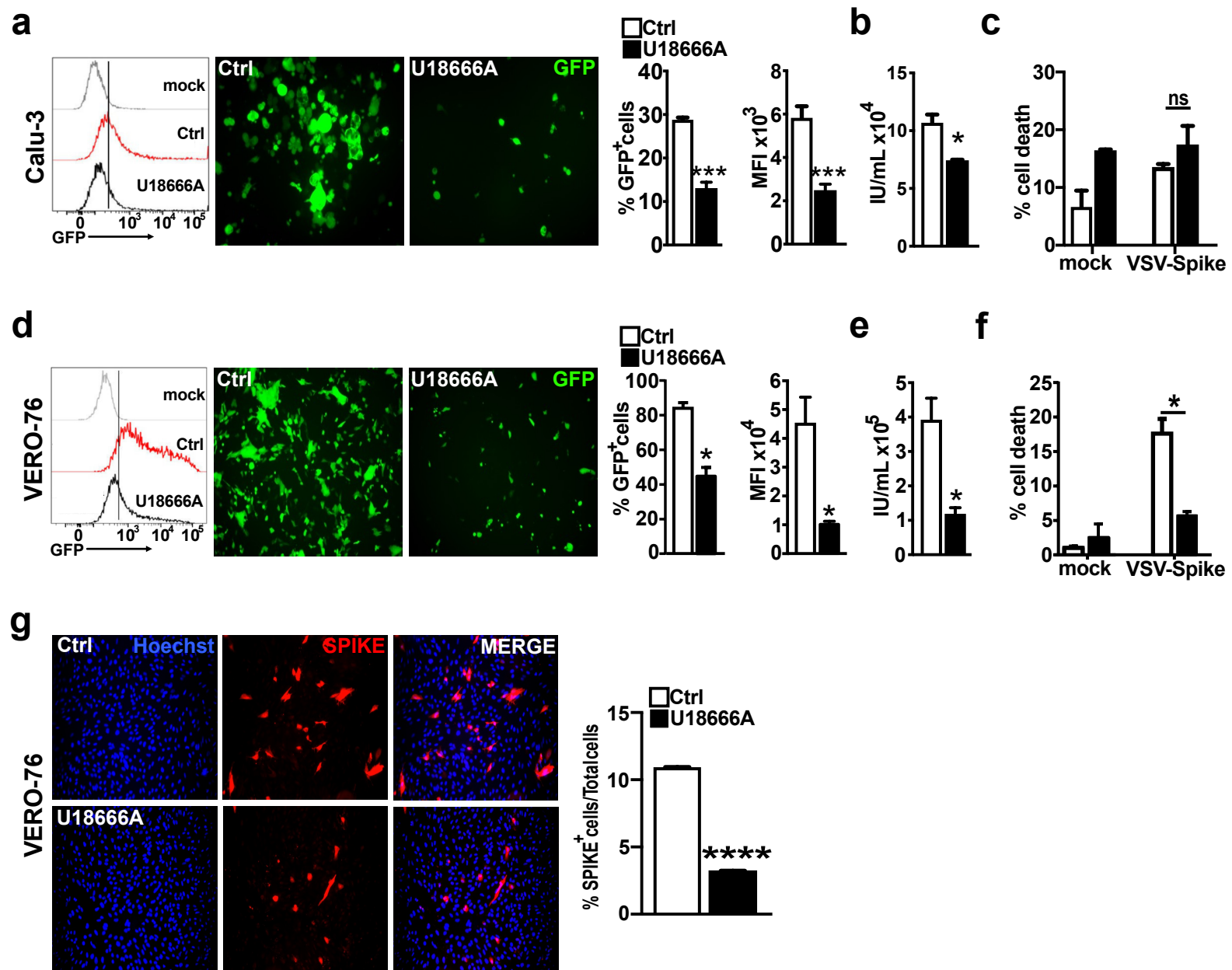

Supplement: Supplementary file 4 — Supplementary Material 4. [file 13578_2024_1331_MOESM4_ESM.pdf]

Figure 1a

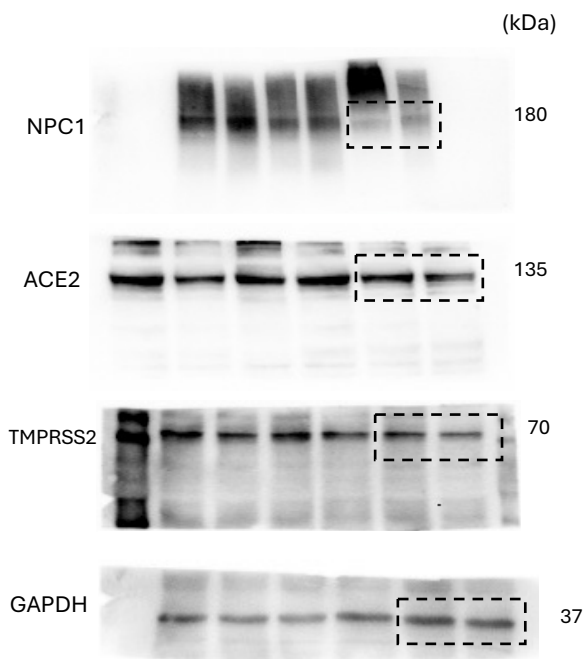

Figure 1b

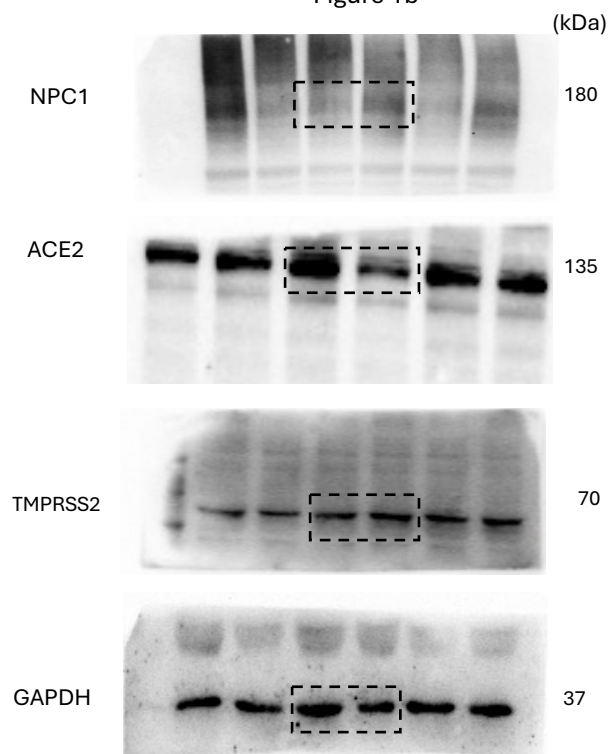

Figure 1c

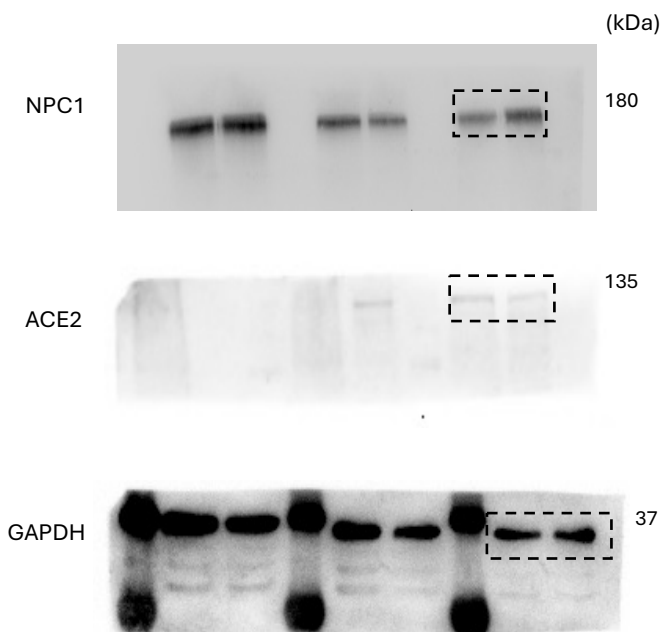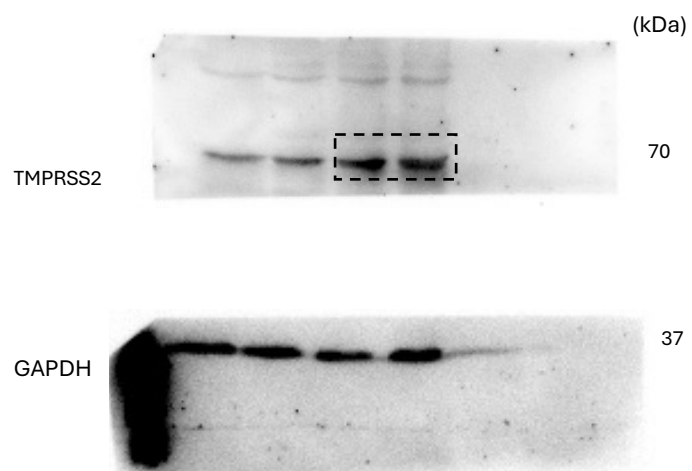

Figure 2f

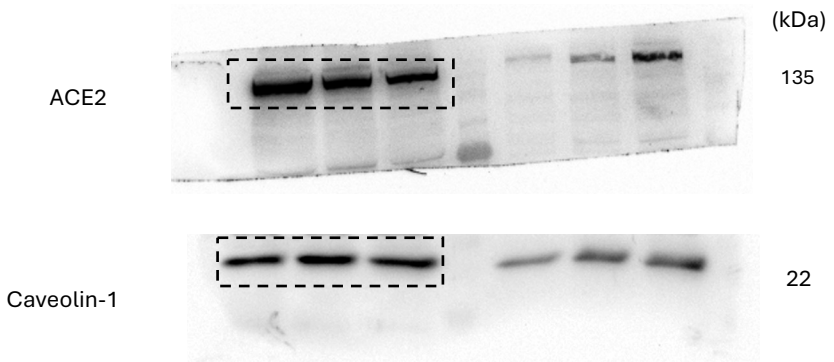

Figure 4b and figure 6b

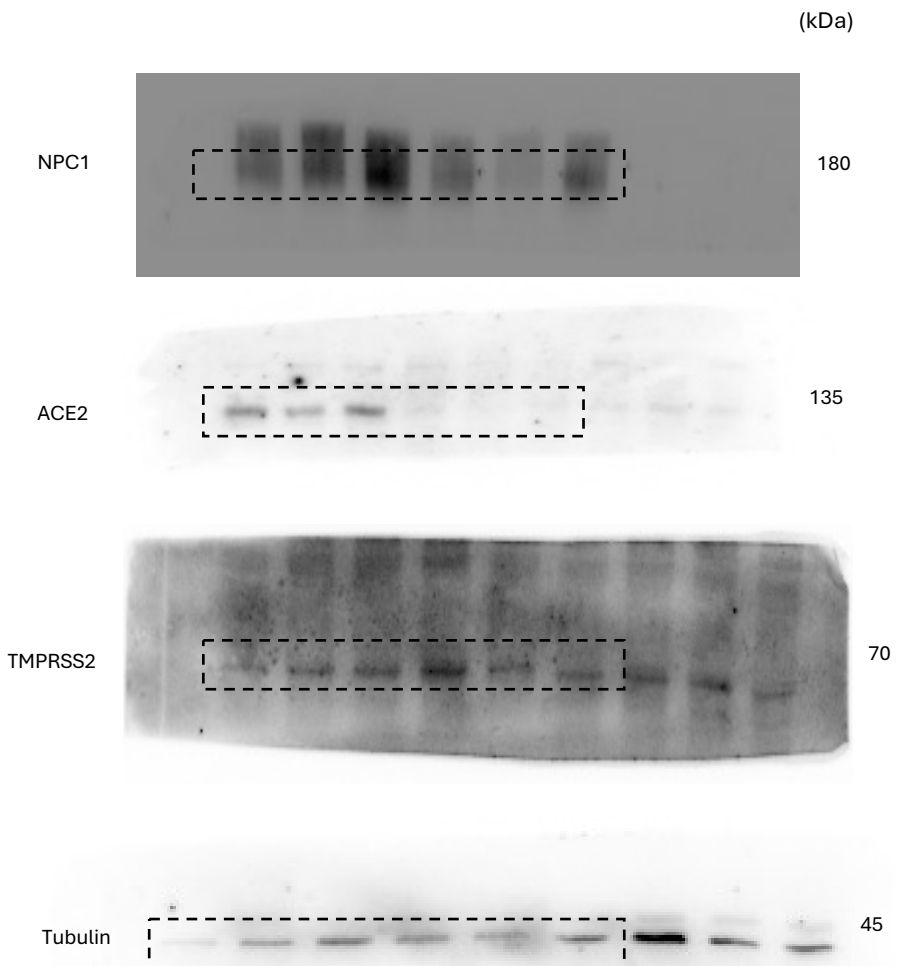

Figure 6d

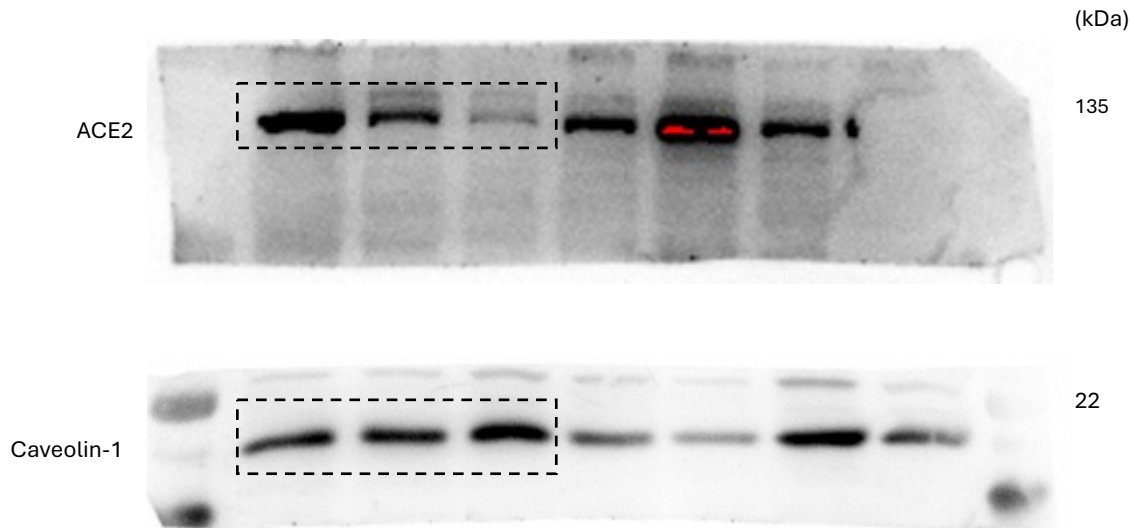

Supplementary fig. 1

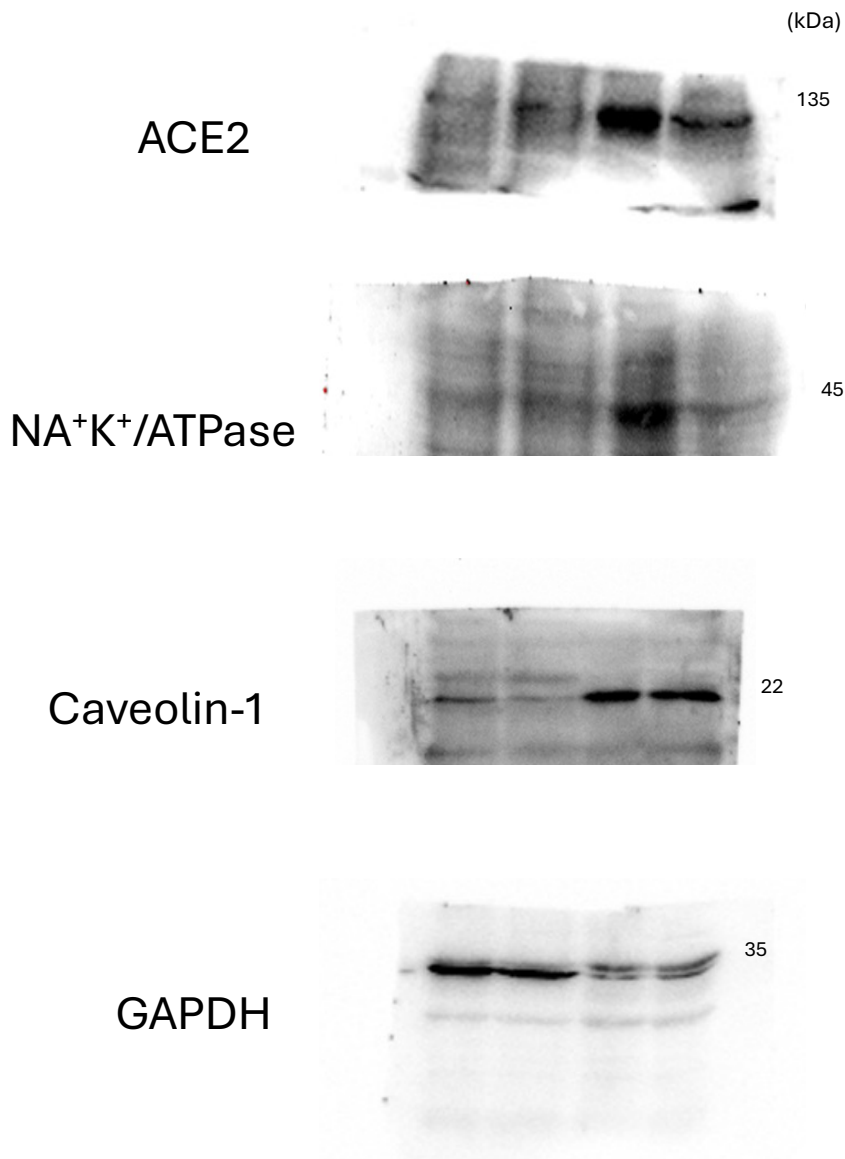

Supplement: Supplementary file 6 — Supplementary Material 6. [file 13578_2024_1331_MOESM6_ESM.pdf]
