# Supplementary material for: The inactivation of the Niemann Pick C1 cholesterol transporter restricts SARS-CoV-2 entry into host cells by decreasing ACE2 abundance at the plasma membrane
Source: Cell Biosci. 2024 Dec 20;14:148. doi: 10.1186/s13578-024-01331-4 (PMC11662611; doi:10.1186/s13578-024-01331-4)
Supplement: Supplementary file 5 — Supplementary Material 5. [file 13578_2024_1331_MOESM5_ESM.docx]

The inactivation of the Niemann Pick C1 cholesterol transporter restricts SARS-CoV-2 entry into host cells by decreasing the abundance of ACE2 at the plasma membrane.

Piergiorgio La Rosa1,2,§, Jessica Tiberi1,3,§, Enrico Palermo4,§, Roberta Stefanelli1,2, Sofia Matia Luigia Tiano5, Sonia Canterini1,2, Mirko Cortese5,6, John Hiscott4, Maria Teresa Fiorenza1,2,*

*§ Equal contribution*

*Corresponding.

**Email:**  [mariateresa.fiorenza@uniroma1.it](mailto:mariateresa.fiorenza@uniroma1.it)

**SUPPLEMENTARY MATERIALS**

**Supplementary figure legends**

**Figure 1S.** Validation of the experimental approach used to isolate plasma membrane proteins. 40-50^x^10^6^ cells, either untreated or U18666A-treated were processed as described under materials and methods. The panel shows the Western blot analysis of the plasma membrane fractions as compared to total lysates. ACE2, Na+/K+ ATPase and Caveolin-1 were detected in the membrane fraction and were barely detected/below detection in the total lysates. By contrast, the presence of GAPDH landmarked the total lysate fraction, while it was slightly detected in the membrane fraction. Ponceau staining was used as loading control.

**Figure 2S**. Validation of NPC1 phenotype obtained by U18666A drug administration in cell lines originating from various tissues. (a) Vero E6-TMPRSS2, Caco-2 and Calu-3 cells were treated with increasing concentration of U18666A (0.02 µm-20 µm) for 24 h and stained with filipin; control cells were treated with 0.2% DMSO (vehicle, Ctrl). Cell growth analysis at 24, 48, and 72 h following treatment initiation with increasing U18666A concentrations (0.02 µM-200 µM). (b) Vero E6-TMPRSS2, Caco-2 and Calu-3 cells were treated either with 0.2 µM or 2 µM U18666A for 24, 48, and 72 h and imaged following filipin staining. Representative images of three independent experiments are shown in A and B. Scale bar: 50 µM. **p < 0.01, ****p < 0.0001 *vs* Ctrl, calculated by TWO WAY ANOVA.

**Figure 3S.** Characterizing how treatment with U18666A influences the intracellular localization of NPC1 and ACE2. (a) Representative images of immunofluorescence analysis of Caco-2 cells treated with 2 µM U18666A or DMSO (Ctrl) for 24 h and then subjected to double immunofluorescence using α-NPC1 (green) and α-ACE2 (red) antibodies. (b-e) Representative images of immunofluorescence analysis of VERO-76 cells treated with 2 µM U18666A or DMSO (vehicle, Ctrl) for 24 h and then subjected to double immunofluorescence by the incubation with the following pairs of antibodies: α-NPC1 (green), α-LC3B (red) (a), α-NPC1 (green), α-LAMP2 (red) (b); α-ACE2 (red), α-LC3B (green) (c), α-ACE2 (green), α-LC3B (red) (d). Nuclei were stained with Hoechst 33342. Images are representative of at least three independent experiments Scale bar: 50 µM.

**Figure 4S.** The inactivation of NPC1 in Calu-3 and VERO-76 cells counteracts VSV-Spike entry. (a) Calu-3 cells were treated with U1866A (2 uM) or DMSO (vehicle, Ctrl) for 24 hr. Cells were then infected with VSV-Spike-GFP (MOI 0.1), harvested 24 h after infection and analyzed for GFP expression. A representative output of FACS analysis displaying the fraction of infected, GFP-positive cells/experimental group (a, left). Representative images of fluorescence microscopy analysis of VSV-Spike-GFP infected cells and quantitative analysis of GFP-positive cells (bars). ***p < 0.0001. (b) Control and U18666A-treated cells were infected with VSV-Spike-GFP (MOI 0.1). Supernatants were collected after 24 h and used to infect Vero E6 cells; at 7 h post-infection the fraction of GFP-positive cells was quantified by flow cytometry and viral titer, expressed as infection units (IU), was determined. Histograms represent mean ± SD from three independent experiments. *p < 0.0001. (c) Cells were harvested 24 h following VSV-Spike-GFP (MOI 0.1) infection and stained for 7-AAD to quantify the fraction of dead cells by flow cytometry. Histograms represent the mean ± SD from three independent experiments; n.s., non-significant. (d) VERO-76 cells were treated with 2 uM U1866A inhibitor or DMSO (vehicle, Ctrl) for 24 h. Cells were then infected with VSV-Spike-GFP (MOI 0.1), harvested 24 h after infection and analyzed for GFP expression by flow cytometry. A representative output of FACS analysis displaying the fraction of infected, GFP-positive cells/experimental group (d, left). Representative images of immunofluorescence analysis of VSV-Spike-GFP infected cells and quantitative analysis of GFP-positive cells (bars). *p < 0.0001. (e) Control and U18666A-treated cells were infected with VSV-Spike-GFP (MOI 0.1). Supernatants were collected after 24 h and used to infect VERO-76 cells; at 7 h post-infection the fraction of GFP-positive cells was quantified by flow cytometry and viral titer, expressed as infection units (IU), was determined. Histograms represent mean ± SD from three independent experiments. *p < 0.0001. (f) Cells were harvested 24 h following VSV-Spike-GFP (MOI 0.1) infection and stained for 7-AAD to quantify the fraction of dead cells by flow cytometry. Histograms represent the mean ± SD from three independent experiments; *p < 0.0001. (g) Representative images of fluorescence microscopy analysis of VSV-Spike-GFP infected cells processed by immunofluorescence with α-Spike antibodies. Histograms represent the fraction of Spike-positive cells. **** p ≤0.0001. Scale bar: 50 µM.

**Supplementary Table 1.** List of oligonucleotides used as PCR primers.

| OLIGONUCLEOTIDE NAME | SEQUENCE 5’🡪 3’ |
| --- | --- |
| *H. sapiens* RPL34 fw | CCAGCGTTTGACATACCGAC |
| *H. sapiens* RPL34 rev | TGCTTTCCCAACCTTCTTGGT |
| *H. sapiens* NPC1 2^nd^-3^rd^ exons fw | TTCTGGCCCACCAAAACCATT |
| *H. sapiens* NPC1 2^nd^-3^rd^ exons rev | CTGCCGAACATCACAACAGAG |
| *C. sabeus* NPC1 5^th^-6^th^ exons fw | ACCATCACTCCCGTGTTTTCA |
| *C. sabeus* NPC1 5^th^-6^th^ exons rev | GGGCCACAGACAATAGAGCA |
| *H. sapiens* ACE2 fw | TCACGATTGTTGGGACTCTGC |
| *H. sapiens* ACE2 rev | CCACCACCCCAACTATCTCTC |
| *C. sabeus* ACE2 fw | TCACGATTGTTGGGACTCTGC |
| *C. sabeus* ACE2 rev | CCACCACCCCAACTATCTCTC |
| *H. sapiens* TMPRSS2 fw | GGAGGACGAGAATCGGTGTG |
| *H. sapiens* TMPRSS2 rev | TCGTTCCAGTCGTCTTGGC |
| *C. sabeus* TMPRSS2 fw | ACTGCTGGATTTCTGGGTGG |
| *C. sabeus* TMPRSS2 rev | AGATCATGGCTGGTGTGACC |
|  |  |
| NPC1 4^th^ exon fw | TGACATGTAGCCCTCGACAG |
| NPC1 4^th^ exon rev | CTGTCCGACGTAGTATTGTAACTC |
|  |  |
| NPC1 4-5^th^ exons fw | TGACATGTAGCCCTCGACAG |
| NPC1 4-5^th^ exons rev | CATCCCGGCAGGCATTGTA |
